# Rainfall trend and variability in Southeast Florida: Implications for freshwater availability in the Everglades

**DOI:** 10.1371/journal.pone.0212008

**Published:** 2019-02-12

**Authors:** Anteneh Z. Abiy, Assefa M. Melesse, Wossenu Abtew, Dean Whitman

**Affiliations:** 1 Department of Earth and Environment, Florida International University, Miami, FL, United States of America; 2 Water and Environment Consulting, Boynton Beach, FL, United States of America; 3 Southeast Environmental Research Center (SERC), Institute of Water & Environment, Florida International University; Universiti Sains Malaysia, MALAYSIA

## Abstract

Freshwater demand in Southeast Florida is predicted to increase over the next few decades. However, shifting patterns in the intensity and frequency of drought create considerable pressure on local freshwater availability. Well-established water resources management requires evaluating and understanding long-term rainfall patterns, drought intensity and cycle, and related rainfall deficit. In this study, the presence of rainfall monotonic trends was analyzed using linear regression and Mann–Kendal trend tests. Pettit's single point detection test examined the presence of an abrupt change of rainfall. Drought in Southeast Florida is assessed using the Standardized Precipitation Index (SPI) in 3-, 6-, 12-, and 24-months scale; and the Fast Fourier Transform is applied to evaluate the frequency of each drought intensity. There was an increase of rainfall in most of the wet season months, the total wet season, and the annual total. The wet season duration showed a decrease driven by a decrease in October rainfall. Since 1990, wet season and total annual rainfall exhibited an abrupt increase. The SPI analysis has indicated that extended wetness characterizes the contemporary rainfall regime since 1995, except for the incidence of intermittent dry spells. Short-term droughts have 3-year to 5-year recurrence intervals, and sustained droughts have a 10-year and 20-year recurrence intervals. In Southeast Florida, prolonged drought limits freshwater availability by decreasing recharge, resulting in a longer hydro-period to maintain the health of the Everglades Ecosystem, and to control saltwater intrusion. The increasing dry season duration suggests the growing importance of promoting surface water storage and demand-side management practices.

## Introduction

The increasing incidence of extreme hydroclimatic conditions, particularly drought, plays a vital role in regulating water resource availability. In Southeast Florida, rainfall is a significant component of the local hydrologic budget [1,2], with rainfall deficit limiting surface and groundwater availability. As water resources are completely allocated, any change in the precipitation regime has a substantial impact on the local water management system [1]. Incidence of drought in the area forces water managers to revise water allocation practices and management criteria that are specific to drought. However, these revisions do not present long-term solutions. Improving freshwater availability in the area requires preparedness for water resource planning, and management in the face of drought.

Natural hydroclimate variability prompts a cascade of adverse effects caused by the reduced availablity of atmospheric moisture, resulting in meteorological drought [3–6]. Meteorological drought decreases soil water content, promoting slow plant growth, and directly affecting agricultural productivity (agricultural drought). Extended atmospheric dryness results in a prolonged but small-scale rainfall deficit [7,8], which diminishes surface water flow, groundwater recharge, and water in storage (hydrological drought).

In Southeast Florida, incidence of drought has historically caused a decrease in surface water levels [2,9,10]. As surface water level decreases, groundwater recharge decreases, leading to groundwater level decline, which can trigger saltwater intrusion [9–11]. Ultimately, drought in the area creates an intricate challenge that limits freshwater availability, threatening the Everglades’ vulnerable Ecosystem [12,13]. To curb the adverse effects of drought on the hydrological system, we must understand the evolution of drought in the area by analyzing and evaluating long-term historical rainfall trends and reconstructing historical drought cycles. Such an understanding can enhance our knowledge of the local drought variability at a different time domain, a key input to long-term resources allocation and strategic planning. Because the study of drought’s hydrological impact in the area has scarce coverage and is limited to the short-term, it is necessary to conduct long-term drought analysis and indentify implications to current and future water resources availablity.

The objectives of this study are to 1) evaluate long-term (111 years) rainfall patterns, 2) describe historical drought conditions at different time scales, and 3) assess the frequency of different classes of drought in Southeast Florida. We have analyzed historical regionalized monthly rainfall data in Southeast Florida from 1906 to 2016. For such long-term historical hydrological drought evaluation, data on parameters such as evapotranspiration and soil moisture are scarce. Instead, we consider drought to be a system continuum whose effect propagates from meteorological drought to hydrological drought [4,5,9,10] and evaluate the local drought using the Standardized Precipitation Index (SPI). Drought frequency is examined by using a Fast Fourier Transformation (FFT). Based on the overall analyses, we have evaluated and presented potential implications for regional freshwater availability.

### Description of the study area

**Fig 1:**
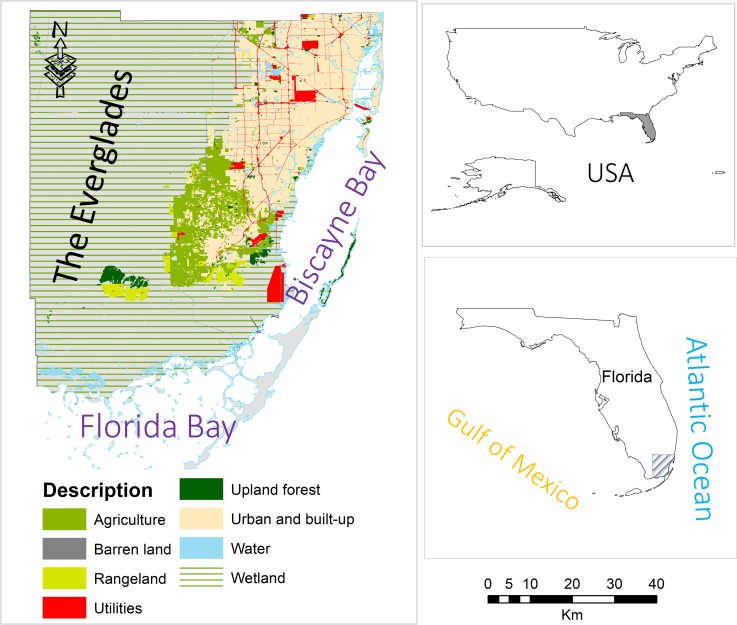
Location map of the study area. Cartographic Boundary Shape files are freely available at US Census Bureau.

The Southeast Florida peninsula has a flat topography that rises from 0.0 m to 7.0 m above NAVD 88. The average elevation in the area is 1.5m (Fig 1). Around 25% of the area is urbanized land cover with predominantly impervious surface. The Everglades wetland covers most of the study area, extending from the water conservation area southeast of Lake Okeechobee to the Florida Bay [12–15].

In Southeast Florida, the primary source of fresh water for more than 6 million people is the Biscayne Aquifer [16]. Surface water in lakes and the Everglades helps to maintain high groundwater head and control saltwater intrusion by providing groundwater recharge. The freshwater in the area is optimally allocated, and any shift in the hydrologic regime requires considerable readjustment on water allocation and management criteria [14–17]. Increased groundwater pumping, the recurrence of drought, and sea level rise threaten freshwater availability in Southeast Florida [9,15].

### Hydrometeorology

**Fig 2:**
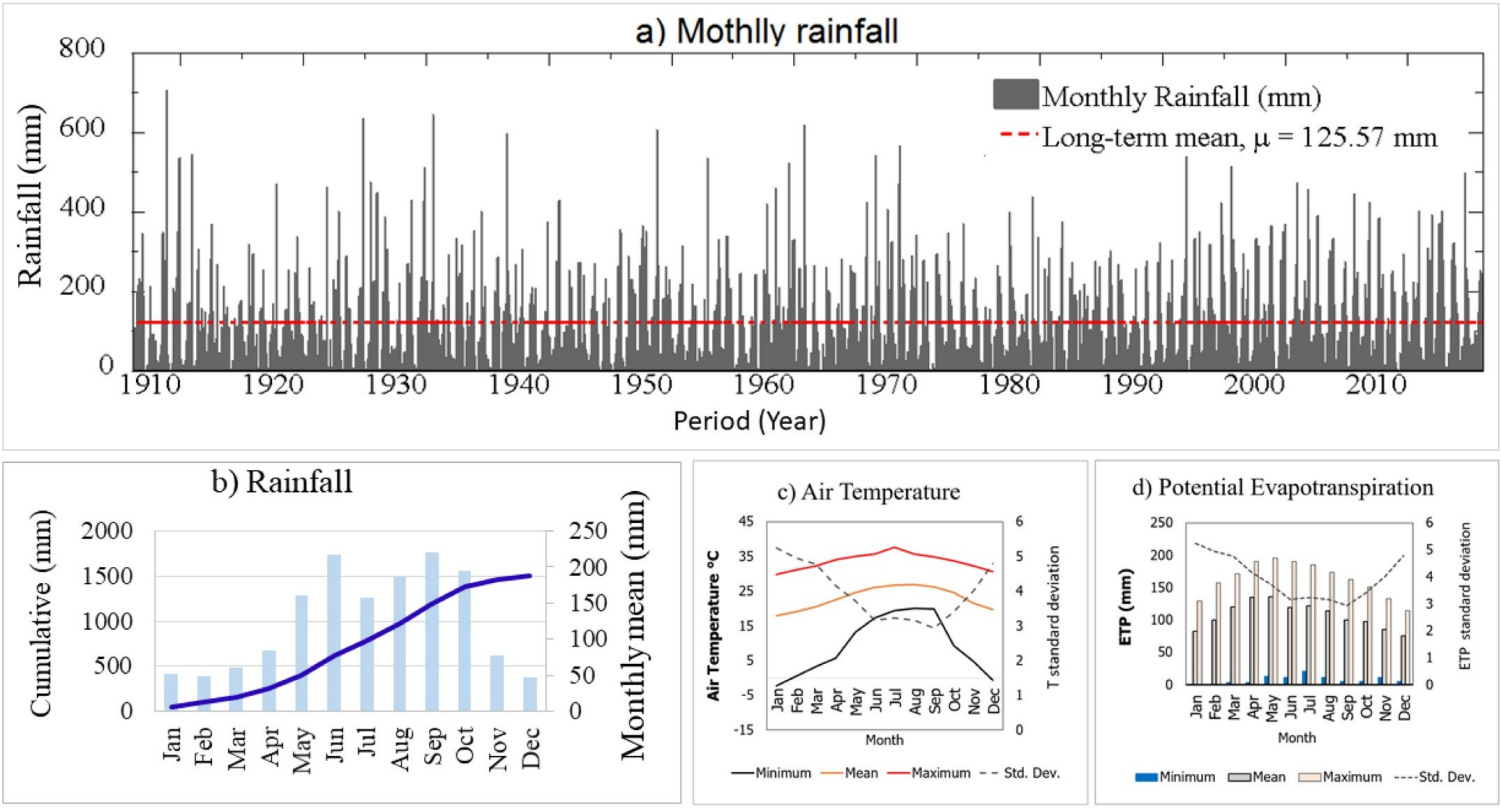
(a) Monthly rainfall distribution in Southeast Florida from 1906–2016, (b) Histogram and cumulative rainfall monthly rainfall of Southeast Florida (1906–2016), C) Monthly composite air temperature, and d) potential evapotranspiration. Monthly composite data for air temperature and ETP are collected from S331W climatological measurement station of the South Florida Water Management District.

Southeast Florida has a tropical monsoon climate characterized by hot and humid rainy summer, and mild winters. The annual average minimum, mean, and maximum temperatures in the area are 15°C, 26°C, and 32°C, respectively. A wide range of temperature with standard deviation of 3°C -6°C is recorded in the area. Southeast Flori has a bi-modal rainfall distribution with a long-term total annual average of 1507 mm. The total annual average potential evapotranspiration (ETP) loss in the area ranges from 1220 mm to 1320 mm per year (Fig 2).

Rainfall in Southeast Florida is highly variable and is driven by local and regional hydroclimatic factors [2]. The area receives rainfall from convective, tropical cyclone, and frontal rainfall systems [18]. Its topography, geographic location adjacent to the ocean, and degree of urbanization has significant effect on the spatial and temporal distribution of rainfall. A wide range of precipitation intensity characterizes the warm and humid summer season. There is a high range of variation in local long-term rainfall between months within a year. Likewise, specific months show a wide range of rainfall variation across different years (Table 1). Overall, the hydrologic regime in the area is divided into dry (November through April) and wet seasons (May through October) (Fig 2).

**Table 1:** Descriptive statistics of mean monthly rainfall record from 1906 to 2016; where, STDEV and CV stand for the standard deviation and coefficient of variation, respectively.

Description	Dry						Wet					
Month	Nov	Dec	Jan	Feb	Mar	Apr	May	Jun	Jul	Aug	Sep	Oct
Mean Rainfall (mm)	77.2	46.9	52.0	48.9	60.5	85	160.8	217	157.4	186	220.4	194.6
STDEV (mm)	75.1	45.9	45.2	39.1	54.4	73.3	106.3	125.3	69.2	84.8	113.1	145.3
CV (%)	97.3	97.8	86.6	80	90	86.2	66.1	57.7	44	45.6	51.3	74.7

Around 75% of the total precipitation falls in the wet season, which comes from convective rainfall and tropical systems. The dry season rainfall is from frontal systems. Discharge from regional groundwater circulation and controlled release from upstream canals provid a considerable volume of freshwater input to the water balance in the area [1].

Extreme hydroclimatic conditions such as heavy rainfall leading to flooding, and precipitation deficit causing drought, are common in the Southeast Florida. Sustained decline of rainfall below the long-term normal was reported in 1932,1955–57,1961–63, 1971–72, 1973–74, 1980–82, 1985, 1988–89, 1990, 2000–2001, 2006–2007, 2011–2012 [1,2]. These drought events have a strong association with the La-Nina phase of the ENSO events [19]. When drought occurs, a decline in surface water level is observed in reservoirs, such as Lake Okeechobee. Furthermore, increasing intensity and duration of drought have caused a significant disturbance in the Everglades ecosystem [2,12,20]. The increasing incidence of drought is a critical challenge to the current and future water resources management in Southeast Florida, by decreasing freshwater supply while demand for freshwater in the area will remain high.

## Dataset and methodology

### Rainfall data

Regional monthly rainfall data from 1906 to 2016 was obtained from the Florida Climate Center (FCC) (http://climatecenter.fsu.edu/products-services/data/precipitation/miami), who acquired their data from the National Weather Service's Cooperative Observation (NWSCO) network and the Automated Surface Observing System (ASOS). The rainfall station at Miami International Airport (MIA) was the most comprehensive data source used by the FCC to develop the regional monthly rainfall data. NWSCO and ASOS data fill the gaps left by the MIA rainfall records. Studies in South Florida indicate that a station can represent the monthly regional rainfall within 80 km distance [18,21]. Therefore, this data is representative of Southeast Florida regionalized rainfall.

### Rainfall trend evaluation

The rainfall trend was evaluated in three categories. The first group is the sequential time series rainfall data based on a month-by-month record. The second category is the analysis of interannual variability which uses annual maximum, minimum, and mean, and the total rainfall of wet (MJJASO) and dry (NDJFMA) seasons. The last category constitutes evaluation of the total yearly rainfall trend.

Monotonic and stepped trend detection approaches evaluate rainfall trends. The presence of a rainfall monotonic trend was examined using linear regression and Mann–Kendal (MK) trend tests [22–24]. The linear regression test has the advantage of evaluating linear temporal trends. The t-test statistics evaluate the statistical significance of the monotonic trend defined by linear regression. The MK test is a robust non-parameter, ranked, monotonic trend test that indicates the presence of an upward or downward trend. Z- score is used to assess the significance of the trend detected by MK test. The rate of change of the trend detected by the MK test is calculated using the Sen-Theil trend line slope estimate (Sen's slope). Sen's slope defines the rate of long-term rainfall data’s monotonic trend [25–28]. The details of the algorithms for the MK test and Sen's slope are well explained elsewhere [29]. Pettitt's single point change detection test assesses the presence of an abrupt change of the mean of the long-term rainfall record [25,29–31]. The detection test estimates the change of the mean between successive points in the sequential time series data [32]. All the analysis is calculated at a 95% confidence interval.

### Drought evaluation

The historical drought in Southeast Florida was evaluated using two approaches: an analysis of long-term rainfall residual of monthly rainfall and drought evaluation with SPI [33,34].

#### Drought evaluation with rainfall residual analysis

The difference of each month’s rainfall record from the long-term mean of each corresponding month is calculated to evaluate the long-term monthly rainfall residual. The residual represents the overall wetness or dryness of each month as compared to the long-term mean of each corresponding month. With this approach, a year-month matrix of the rainfall residual is interpolated by using Empirical Bayesian Kriging (EBK) package in ArcGIS 10.4. EBK was selected because it includes more accurate standard errors of forecasting by developing an interpolated surface. Geostatistical evaluation by kriging is among the best interpolation methods for evaluating monthly rainfall distribution in South [18,21,35].

#### Drought evaluation with Standardized Precipitation Index (SPI)

The regionalized rainfall record from 1906 to 2016 is used to compute SPI [33,34,36]. SPI can establish the incidence of drought using rainfall data and has the flexibility to calculate droughts in different time windows, such as 3-, 6-, 12-, and 24-months. Therefore, it can effectively evaluate drought duration and magnitude. SPI results can be used to establish the frequency of different intensities of drought [36,37]. Use of 30 to 50 years of monthly information is recommended for SPI-3 to SPI-24 analyses. For drought evaluation over a longer time window, SPI-24 or longer, more than 60 years of data is recommended [36]. SPI calculation requires normally distributed rainfall data. However, long-term monthly rainfall data is not normally distributed. For rainfall data with a gamma probability distribution function, an approach to estimating SPI by using cumulative probability is analytically solved [33,34] with the following equation:

$$\mathrm{SPI}=\{\begin{array}{c} -\left(t-\frac{c_{0}+c_{1}+c_{2}t^{2}}{1+d_{1}t+d_{2}t^{2}+d_{3}t^{3}}\right),\\ t=\sqrt{\ln\left(\frac{1}{H{\left(x\right)}^{2}}\right)},\;0<H\left(x\right)\leq 0.5\\ t-\frac{c_{0}+c_{1}+c_{2}t^{2}}{1+d_{1}t+d_{2}t^{2}+d_{3}t^{3}},\\ t=\sqrt{\ln\left(\frac{1}{1-H{\left(x\right)}^{2}}\right)},\;0.5<H\left(x\right)<1 \end{array}$$ (1)

where x is a continuous random variable, and in this case, x is the observed rainfall data in a given time window (the running sum in 3-,6-,12-, and 24-moths time window). H(x) is a modified cumulative probability as a function of the probability of no rainfall at x = 0, and the constant values are given as:

$$c_{0}=2.515517,\;c_{1}=0.802853,\;c_{2}=0.010328,$$

$$d_{1}=1.432788,\;d_{2}=0.189269,\;d_{3}=0.001308.$$

The equation is valid only if the rainfall has a gamma probability distribution [37]. For effective implementation of the above equation, evaluation of the rainfall data distribution is required. A MATLAB function that solves Eq (1) is used to evaluate SPI in 3-, 6-, 12-, and 24-months.

#### Drought severity analysis

Drought severity (D_s_) is a measure of the continuation of an event drought over time. In this case, D_s_ is the cumulative negative SPI values, given as:

$$D_{s}=\sum_{i=1}^{n}SPI_{i}\;\forall SPI<0$$ (2)

where D_s_ is the drought severity, n is drought duration, and i is SPI values of successive months of the given SPI-x such that the SPI is negative.

#### Drought frequency analysis

The Fourier transform is used to calculate the frequency of wet-dry cycles evaluated by SPI-x [38]. For such hydrological data with equal sampling interval and specific period, the Discrete Fourier Transform (DFT) frequency analysis is commonly applied [38,39]:

$$X_{j}=\sum_{k=0}^{N-1}x_{k}e^{-i\frac{2\pi }{N}kj}$$ (3)

$$k=0,1,\ldots ,N-1;\;j=0,1,\ldots ,N-1;\;i=\sqrt{-1}$$

where X_j_ is the j^th^ discrete frequency component of the SPI-x time sequence X_k_, and N is the total numbr of data.

Alternatively, the transform result can be interpreted by using the amplitude spectra or power spectral density (PSD). The amplitude spectra is a normalized absolute value of the complex array, and the PSD is a standardized product of the frequency-domain signal and its complex conjugate, given as:

$$A=\frac{\left|X_{i}\right|}{N},\;\mathrm{and}$$ (4)

$$P_{j}=\frac{X_{j}X_{j}^{*}}{N*N}=\frac{{\left|X_{j}\right|}^{2}}{N^{2}}$$ (5)

where A is the scaled amplitude spectra, and P_j_ is the power spectral density.

## Results and discussion

This section contains the trend analyses of monthly, interannual, and yearly rainfall evaluated by linear regression and MK trend tests. We present the results of long-term rainfall homogeneity for seasonal and total annual records, the historical drought evaluation, drought severity, and drought frequency; and we discuss the implications of rainfall trend and drought to freshwater availability.

### Monthly rainfall trend and homogeneity

**Fig 3:**
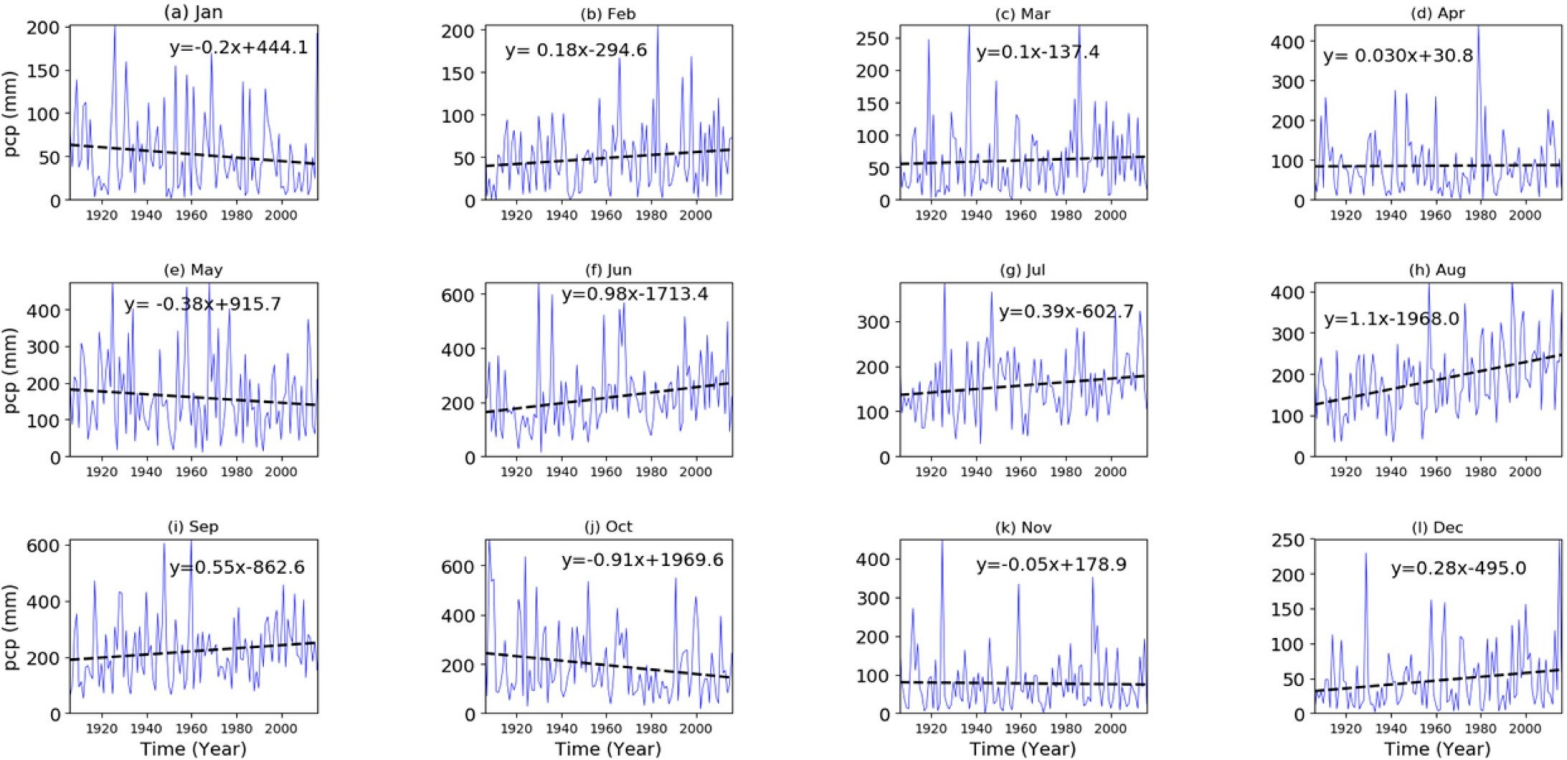
Monthly rainfall regression analysis.

There is a decreasing pattern in the monthly rainfall for October, November, January, and May, and an increasing pattern for February, March, April, July, December, June, August, and September (Fig 3).

However, only the rainfall trends in October, June, and August are statistically significant (Table 2). The decreasing trend in October is 0.91mm/year, while the rainfall for June and August are increasing 0.98 mm/year, and 1.1 mm/year, respectively (Table 2).

**Table 2:** Test statistics of monthly rainfall trend test. For a 95% confidence interval, the computed p-value of less than 0.05 indicates the presence of trend, and the MK test statistics |Z| >1.96 indicates the presence of a significant trend.

Month	Jan	Feb	Mar	Apr	May	Jun	Jul	Aug	Sep	Oct	Nov	Dec
m	-0.2	0.18	0.1	0.03	-0.38	0.98	0.39	1.1	0.55	-0.91	-0.05	0.28
t	-1.50	1.52	0.62	0.13	-1.22	2.73	1.91	4.79	1.66	-2.14	-0.23	2.06
P(t)	0.14	0.13	0.53	0.90	0.22	0.**007**	0.058	< 0.**0001**	0.099	0.**035**	0.82	**0.042**
mu	52	48.9	60.5	85	160.8	217	157.4	185.95	220.4	194.6	77.2	46.9
Kendall's tau	-0.11	0.08	0.08	0.00	-0.08	0.22	0.13	0.27	0.14	-0.09	0.02	0.12
p-value	0.09	0.25	0.21	0.98	0.20	0.00	0.04	< 0.0001	0.03	0.16	0.77	0.05
Z	-1.69	1.16	1.26	-0.03	-1.28	3.50	2.10	4.27	2.21	-1.40	0.30	1.93
Sen's slope	-0.17	0.12	0.01	0.00	**-0.36**	**1.00**	**0.41**	**1.02**	**0.71**	**-0.42**	0.03	0.17
w	3.45	3.25	4.01	5.64	**10.67**	**14.4**	**10.45**	**12.34**	**14.63**	**12.91**	5.12	3.11

m = slope of the regression line (mm/year), mu = average rainfall (mm), and w = rainfall contribution (percent).

The rainfall for June, July, and August showed increases by 1.003mm/year, 0.408 mm/year, 1.025 mm/year, and 0.711 mm/year, respectively. The calculated test statistics of rainfall for June (3.5mm), July (2.1mm), August (4.3mm), and September (2.2mm) indicates the presence of a significantly increasing rainfall trend over the recorded period (Table 2).

The months of May through October contribute the most rainfall to the area (Table 2). Specifically, June and August contribute almost 30% of the total annual precipitation. Hence, an increasing trend of the rainfall for these months can have a significant impact on the total wet season rainfall. Because it is balanced by the increase in earlier months, the decreasing precipitation trend in October might not influence the total annual rainfall but may contribute to the shortening of the overall wet season duration (Table 2).

### Interannual rainfall trend and homogeneity

**Fig 4:**
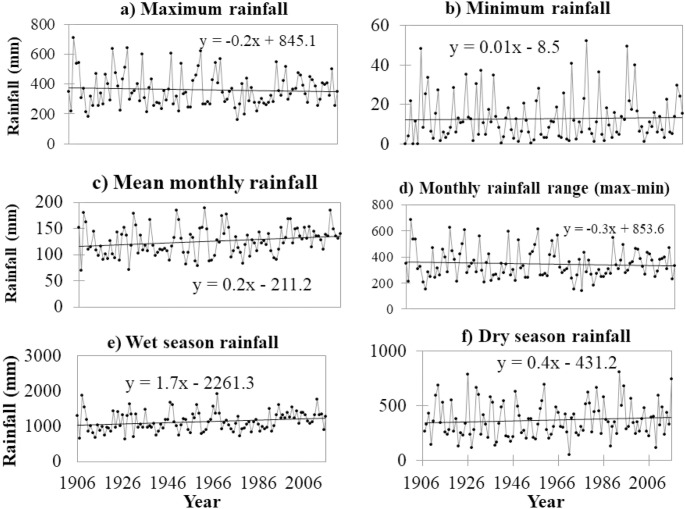
Linear regression of rainfall data: (a) the maximum and (b) the minimum monthly values for each year; (c) The mean monthly values for each year; (d) Rainfall range: the difference between the maximum and minimum monthly value of each year; (e) Wet season rainfall: the sum of the monthly rainfall for May to October; and (f) Dry season rainfall: the sum of rainfall from November to April.

Analysis of the interannual rainfall variabilities showed increasing rainfall trends for the mean annual (t = 2.196, and p (t) = 0.030) and wet season (t = 2.192, p (t) = 0.031) rainfall (Fig 4). The maximum and minimum monthly rainfall records did not exhibit a statistically significant trend.

Under this category, we found a statistically significant trend (Z>1.96) for the annual average (Z = 2.43) and wet season (Z = 2.7) rainfall. The annual average and wet season total rainfall increased by 0.22m/year and 2.23mm/year, respectively.

**Fig 5:**
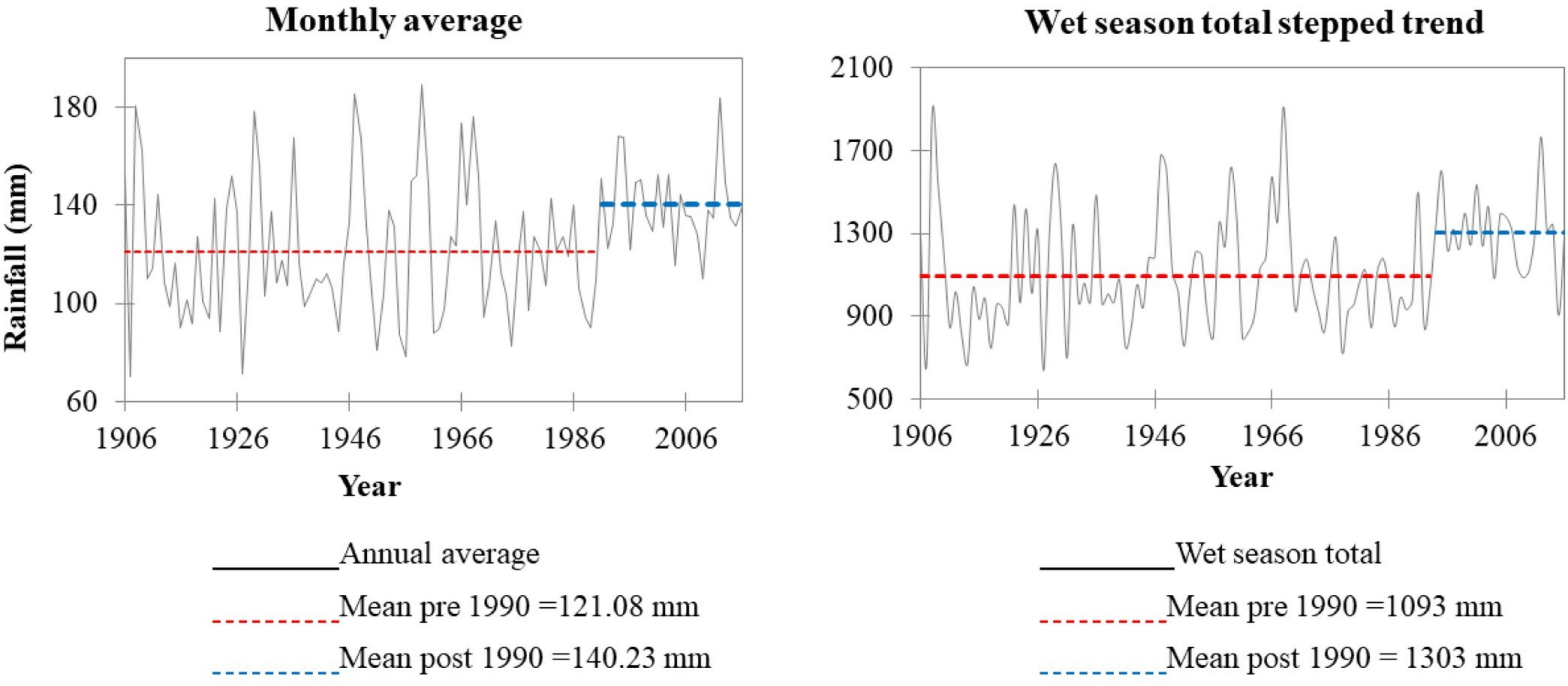
The steeped trend of the long-term monthly average and wet season rainfall. Both have indicated the presence of an abrupt increase of the mean rainfall since 1990.

An abrupt change was spotted for the mean monthly and wet season total rainfall, only. Both changes were spotted in 1990 (Fig 5). The monthly mean exhibited an abrupt change of 15% rainfall increase, and the wet season rainfall showed an increase of 19%.

**Fig 6:**
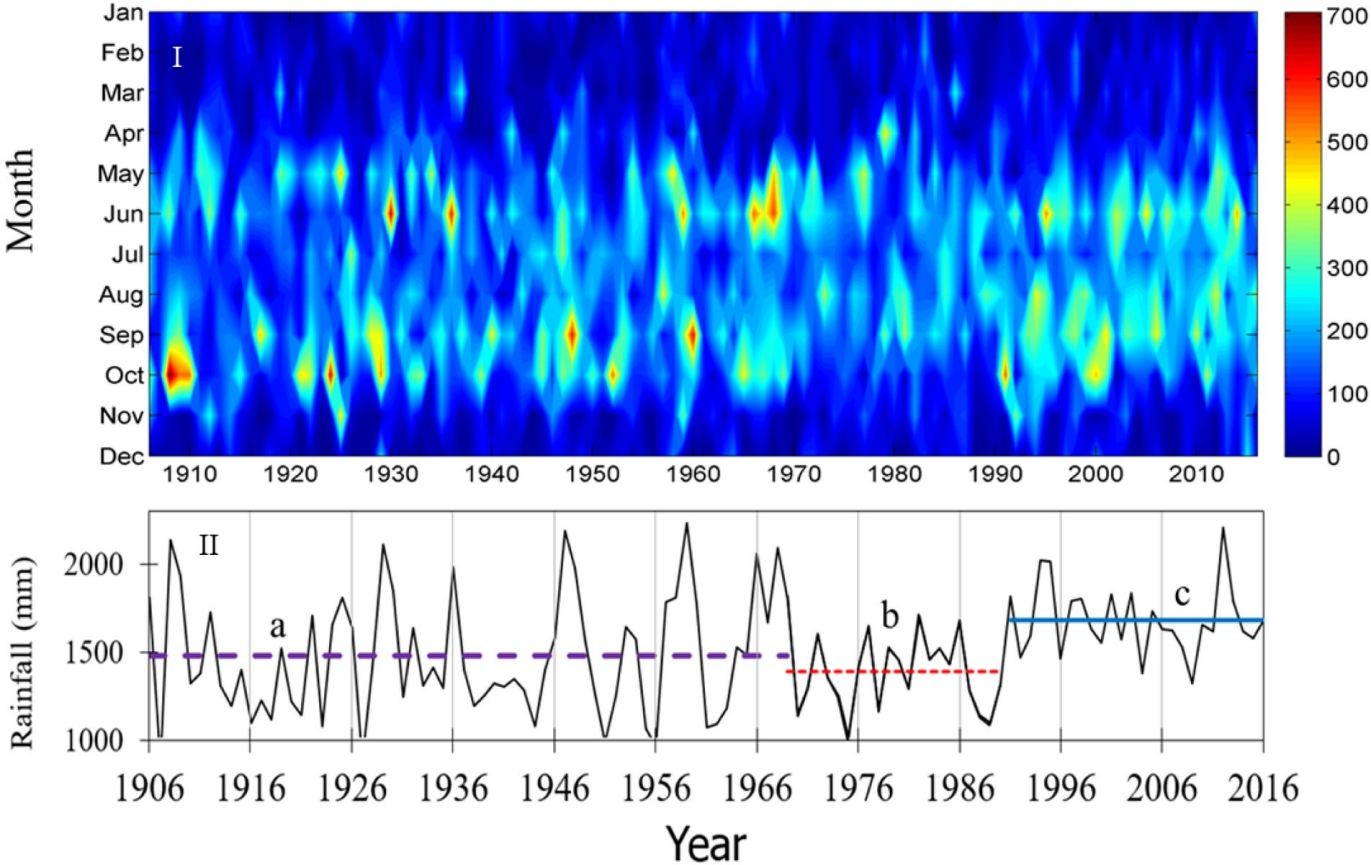
I) Chart of the month-year data matrix and II) stepped trend of the total annual rainfall.

The total annual rainfall is increasing by 2.1mm/year (at t = 2.2, and p (t) = 0.03). An overall increase of the monthly average and wet season total rainfall is attributed to the increase of rainfall in June and August. Like the mean annual and wet season rainfall, the total annual rainfall has shown an abrupt increasing trend since 1990 (Fig 6B). Therefore, we associate the increase in total annual rainfall with the observed increased rainfall for June and August. Interpolation of the observed monthly data as year-month matrix indicates the decreasing trend of the wet season duration (Fig 6A), and this narrowing of the wet season duration is attributed to the decrease for the month of October.

Through the study period (1906–2016), Southeast Florida experienced at least two statistically significant abrupt changes of the mean annual rainfall, spotted in 1969/70 and 1990. The mean rainfall pre-1969 was 1481mm (Fig 6-II-line a). From 1969 to 1990, the mean rainfall dropped to 1368mm (Fig 6-II, line b), and post-1990 it abruptly increased 1685mm (Fig 6-II, line c), accounting for an increase of 16%. This total annual rainfall increase is more or less consistent with the 15% increase of mean monthly rainfall. Although more than 75% of the rainfall in the area is received during the wet season, the post-1990 19% increase of rainfall in the wet seasons has not contributed much to the total annual rainfall increase, which is attributed to the decline of the rainfall in October.

July and August’s rainfall increase and October’s rainfall decline have led to an increasing amount of rainfall and decreasing duration of the wet season. The increasing rainfall in August is gradually altering the rainfall regime from bimodal to unimodal. The abrupt increase of rainfall, the relative narrowing of wet season duration, and the alteration of bimodal rainfall distribution to unimodal distribution are indicated in the year-month matrix chart (Fig 6-I).

### Drought evaluation

#### Drought evaluation with rainfall residual analysis

**Fig 7:**
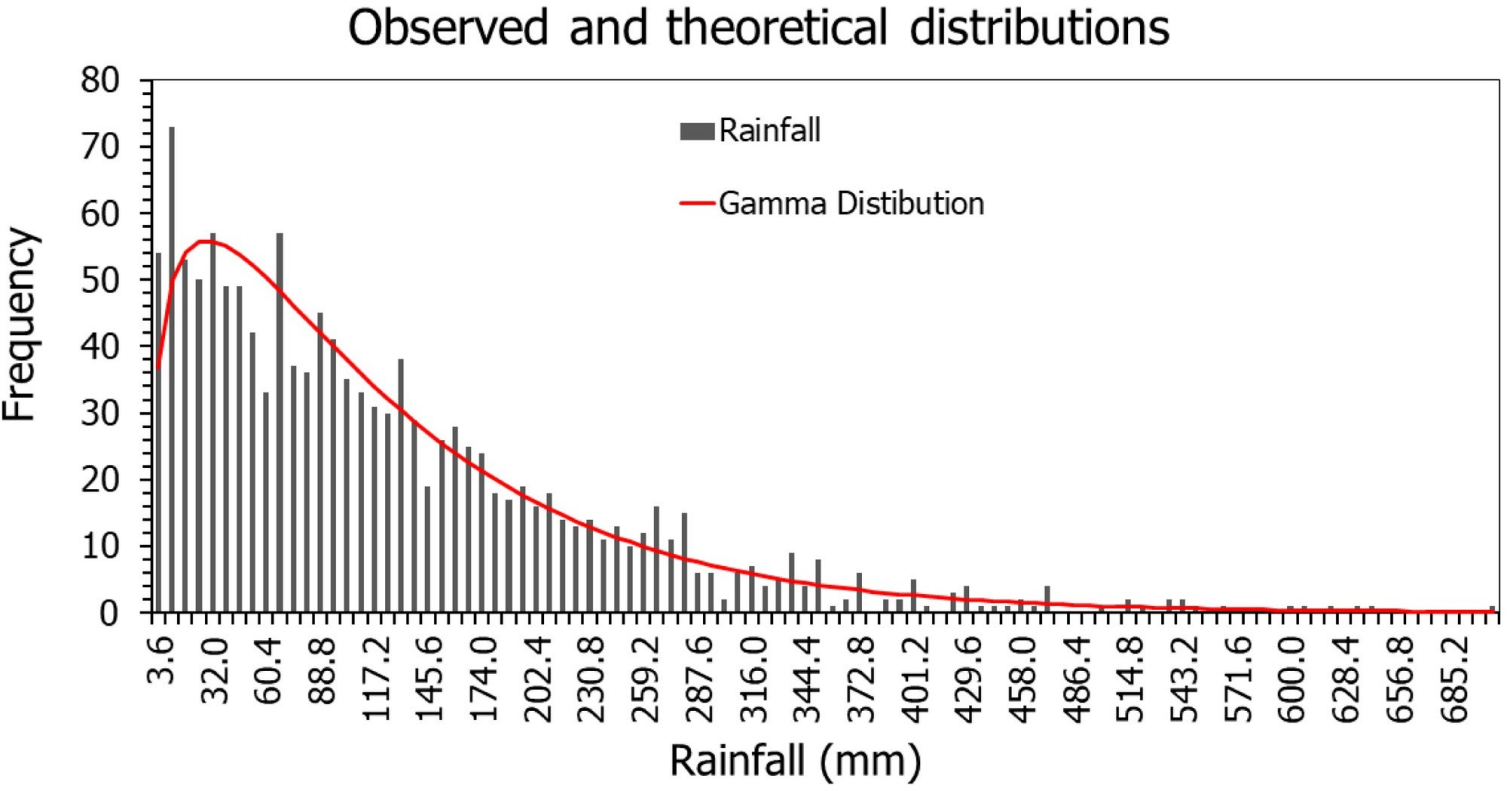
Residual map of monthly rainfall. The deviation of the recorded monthly rainfall from the respective long-term mean monthly value, residual map.

Three distinct patterns of dry and wet fluctuations are portrayed in the chart of the monthly rainfall anomaly (Fig 7). First, it indicates conditions when all months in a year and successive years have received more than average rainfall; second, a pattern when the dry season becomes dryer and the wet season becomes wetter; and third, conditions when all months in a year were dry.

There was a high degree of dryness over a range of years from 1910 to 1925, 1940 to 1947, 1952 to 1958, 1963 to 1966, and 1973 to 1980. Periods from 1908 to 1910, 1928 to 1931, 1945 to 1949, and 1964 to 1969 were wet years. With its distinct fluctuations of the wet and dry phases, the contemporary rainfall pattern in Southeast Florida since 1990 indicates the presence of sustained wetness.

The advantage of the rainfall residual analysis method is that it allows visual interpretation of the rainfall variability in two dimensions, enabling evaluation of the timing and extent of the rainfall deviation from the long-term mean value. The residual map (Fig 7) indicates the magnitude of the monthly rainfall deviation and the temporal propagation of rainfall deficit.

#### Drought evaluation with SPI

**Fig 8:**
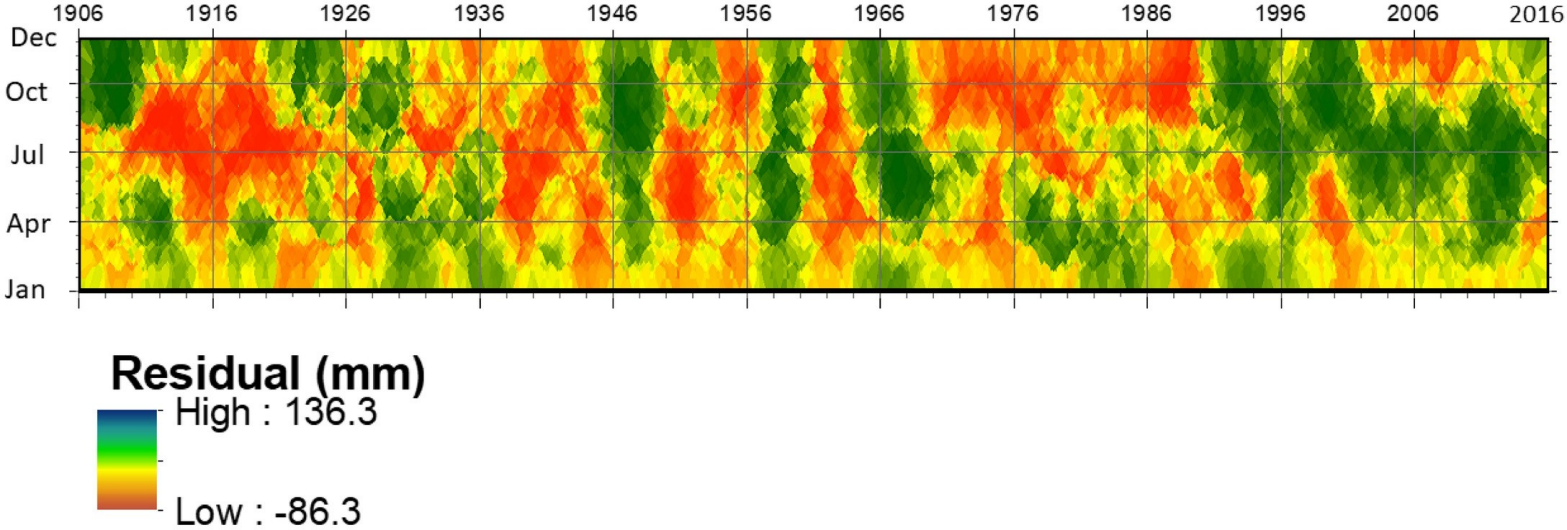
Probability density function and fitted theoretical distribution.

Drought evaluation with SPI begins with defining the probability distribution of the rainfall data. Normality test using Anderson-Darling (A^2^ = 40.9) suggests that the regionalized long-term monthly rainfall data is not normally distributed. Evaluation of the regionalized long-term rainfall data with the gamma probability distribution indicates that the two parameters have positive values (alpha = 1.3 and beta = 96.8), confirming that the rainfall has a gamma distribution (Fig 8). Studies in South Florida indicate that the monthly rainfall in the area follows a gamma distribution [18,21,25]. Therfore, the SPI index can be used to evaluate drought effectively [36].

**Fig 9:**
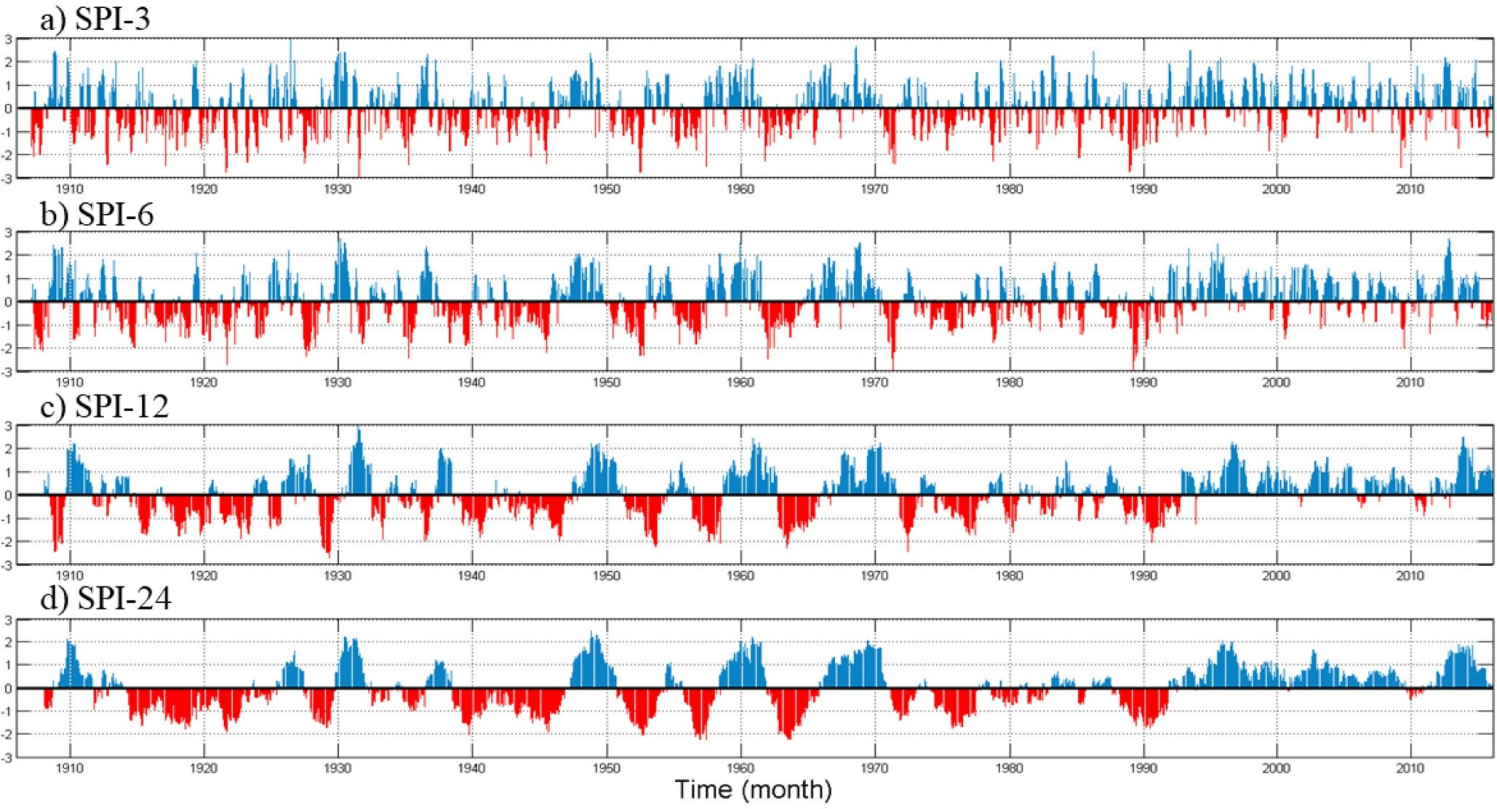
Time series plot of the (a) SPI-3, (b) SPI-6, (c) SPI -12, and (d) SPI-24. The SPI value is the standard normal random variable of the sequential time series data with a positive value indicating wetness, and negative values representing dryness.

The depth of rainfall deficit due to the propagation of drought is evaluated using the SPI in different time windows. SPI value has a mean of zero, and positive (negative) values indicate the presence of wet (dry) total rainfall within the time window under consideration [33, 34]. The different time window SPI (Fig 9) indicated seasonal and long-term precipitation variabilities. The SPI-3 and SPI-6 plots (Fig 9A and 9B) indicate interannual fluctuations, whereas the SPI-12 and SPI-24 (Fig 9C and 9D) indicate the long-term rainfall variability.

Besides the seasonal variations, the SPI-3 and SPI-6 reflect changes in the length of wet and dry seasons within a given year. The SPI-12 and -24 indicated that the area is in the wettest stage of the long-term natural wet and dry cycles.

Apart from incidences of dry spells, Southeast Florida has received prolonged wetness since 1990. However, the SPI analysis indicated that the effect of drought before 1990 was continued until 1994/5. The area has been in an extended wet phase since this time (plots SPI-12 and SPI-24). The prolonged contemporary SPI coincides with the sudden post-1990 increase of the total annual rainfall. Overall, the SPI analysis indicated the temporal propagation of drought, implying a delayed recovery of the hydrologic system after a sustained drought.

**Fig 10:**
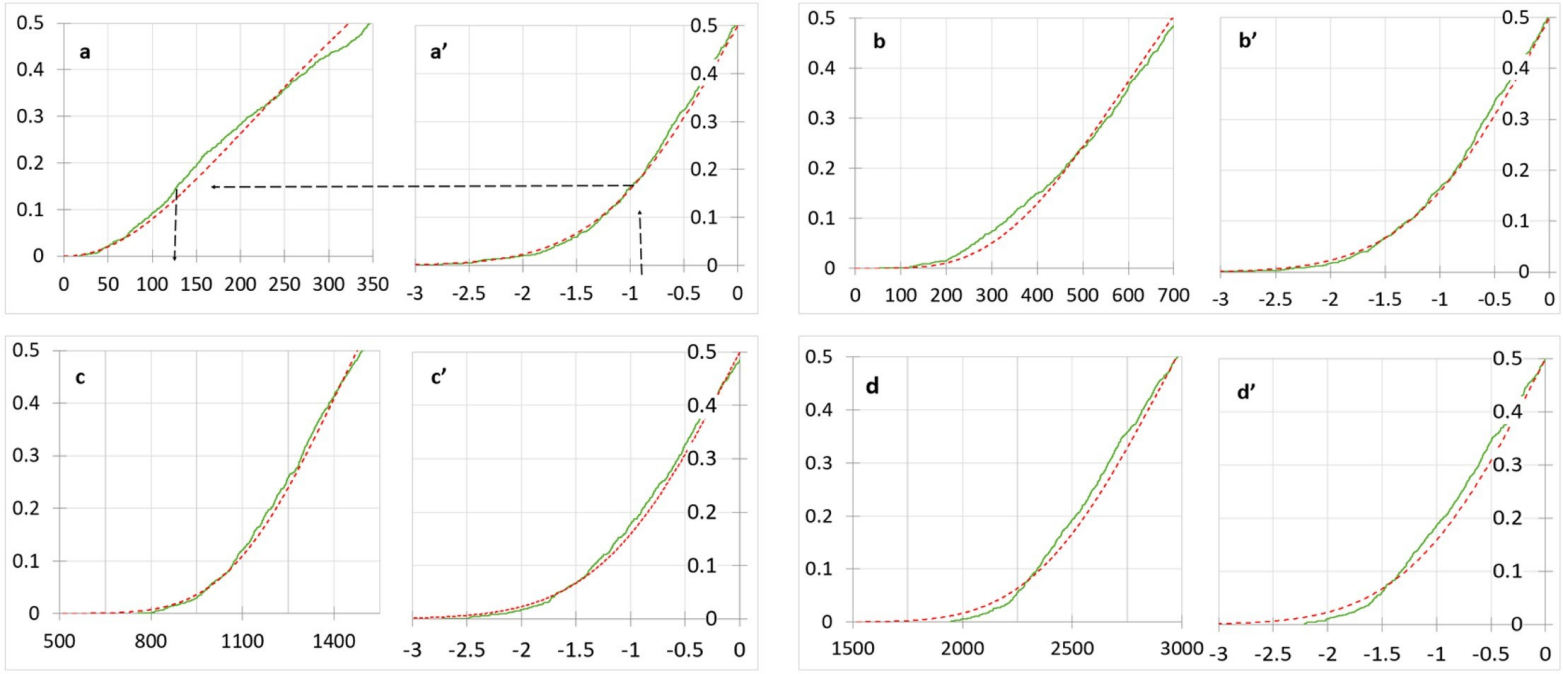
Cumulative histograms for total rainfall and corresponding SPI’s in different time window: a and a’) 3-month total rainfall and SPI-3; b and b’) 6-month total rainfall and SPI-6; c and c’) 12-month total rainfall and SPI-12; and d and d’) 24-month total rainfall and SPI-24.

For a given time window, SPI ≥2.0 is referred to as an extreme wet condition, and SPI value of 1.5 and 1.0 are upper limits for very wet and moderately wet rainfall periods [33,34]. SPI value between 1.0 and -1.0 refers to a neutral condition. SPI value between -1.5 and -1.0 indicates moderate drought, -2.0 to -1.5 indicates severe drought, and less than -2.0 indicates extremely severe drought [33,34]. The rainfall deficit for these drought intensities is computed by combining the probability distributions of the rainfall and the corresponding cumulative distribution SPI value (Fig 10). Hence, the rainfall deficit for a given drought intensity in a specific time window can be outlined by projecting from the cumulative distribution of the SPI to the corresponding cumulative rainfall plot (Fig 10).

The SPI comulative frquancy distribution and the corresponding rainfall cumulative histogram can be used to define the volume of rainfall deficit at different time windows. According to the proposed drought intensity classification using SPI, we tabulate a range of rainfall deficit that corresponds to various intensities of drought in different time windows (Table 3). Such a depth of rainfall deficit refers to the net loss of the total rainfall within the given time window.

**Table 3:** Range of total rainfall deficit (mm) for different drought categories in various SPI time windows.

Drought Category	SPI range	3 months	6 months	12 months	24 months
Moderate drought	-1.5 ≤ SPI < -1	130 to 80	416 to 300	1160 to 1030	2530 to 2290
Severe drought	-2.0 ≤ SPI< -1.5	80 to 56	300 to 232	1030 to 910	2290 to 2040
Extreme drought	SPI < -2.0	>56	> 232	>910	>2040

For drought monitoring and applications to water use, allocation, and management, the relative incidence of rainfall deficit should be monitored in the different time windows. Consideration of drought timing is especially important for rainfall deficit in the 3-month to 6-month time windows. Such droughts make dry seasons drier, and the cumulative effects could pause the hydrological drought. The dryness in the dry period limits the freshwater head in the Everglades. In general, seawater from the ocean intrudes inland during the dry season, and it is discharged back to the sea during the wet season. Monitoring and evaluation results in the Biscayne Aquifer (BA) indicate that only 20% of the intruded water is discharged back into the ocean [40]. The rest remains as a diluted solute in the coastal aquifer. Hence, the repeated occurrence of small-scale dry season droughts can promote a gradual buildup of salinity in the aquifer.

#### Drought severity

**Fig 11:**
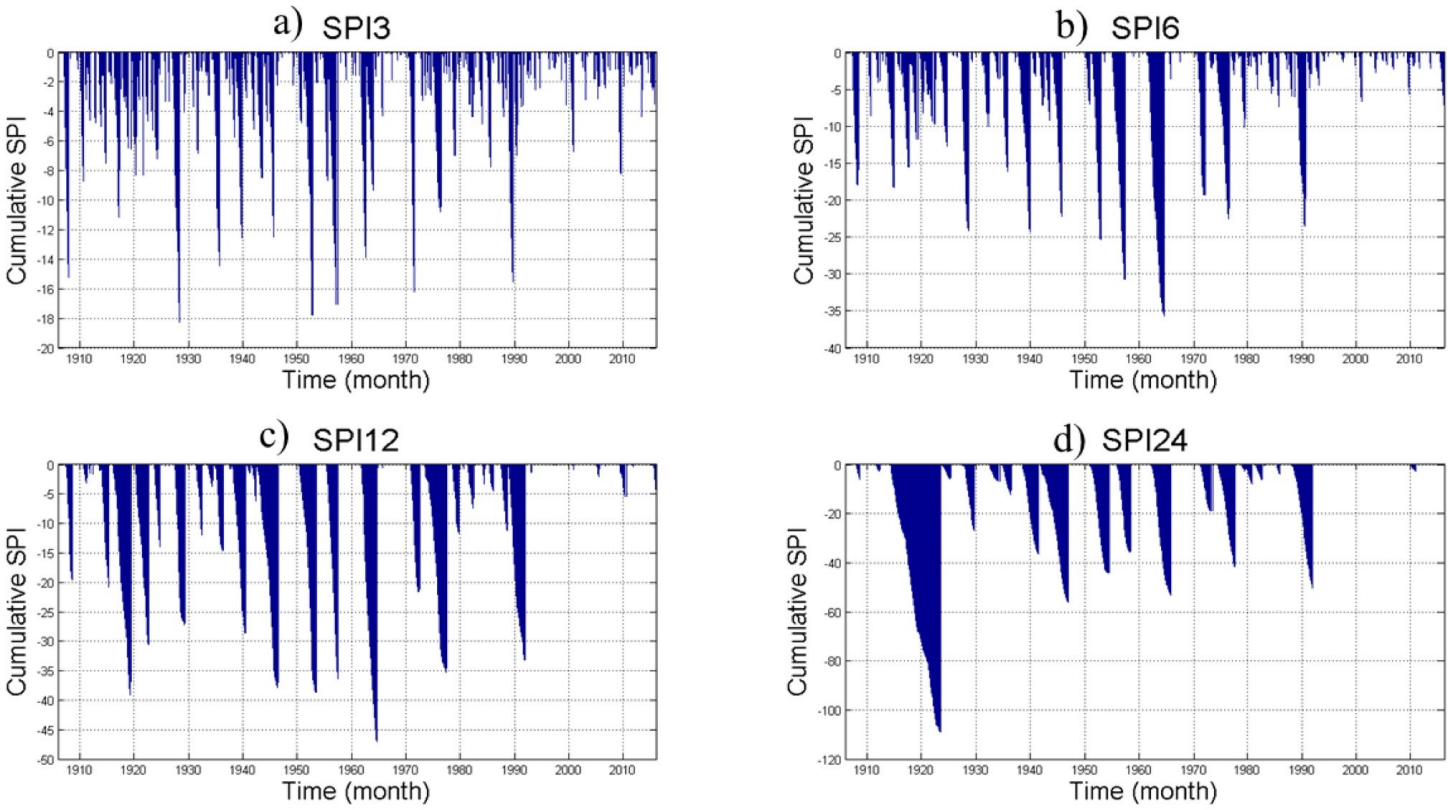
Cumulative values of the (a) SPI-3, (b) SPI-6, (c) SPI-12, and (d) SPI-24.

Hydrological system evaluations should be based on the effect of cumulative rainfall deficit in a given time window [36,37]. Drought intensity is the measure of the cumulative effect of uninterrupted negative SPIs. The study area has seen different phases of drought within various magnitudes and durations (Fig 11).

The drought from 1910 to 2016 is an accumulated long-held but small-scale drought traced by SPI-3. Because the short-term droughts that are shown in SPI-3 from 1990 to 2016 are intermittent, the SPI-12 and SPI-24 plots do not show the incidence of drought in this period. Short ranged, but high magnitude drought events that are recorded by SPI-3 in 1928/29, 1952, 1957, and 1999 have indicated only limited propagation effect in an annual and two-year period. Since the repeated occurrence of small intensity droughts can have a cumulative effect, it potentially causes a significant impact on the local water resource system.

#### Drought frequency

**Fig 12:**
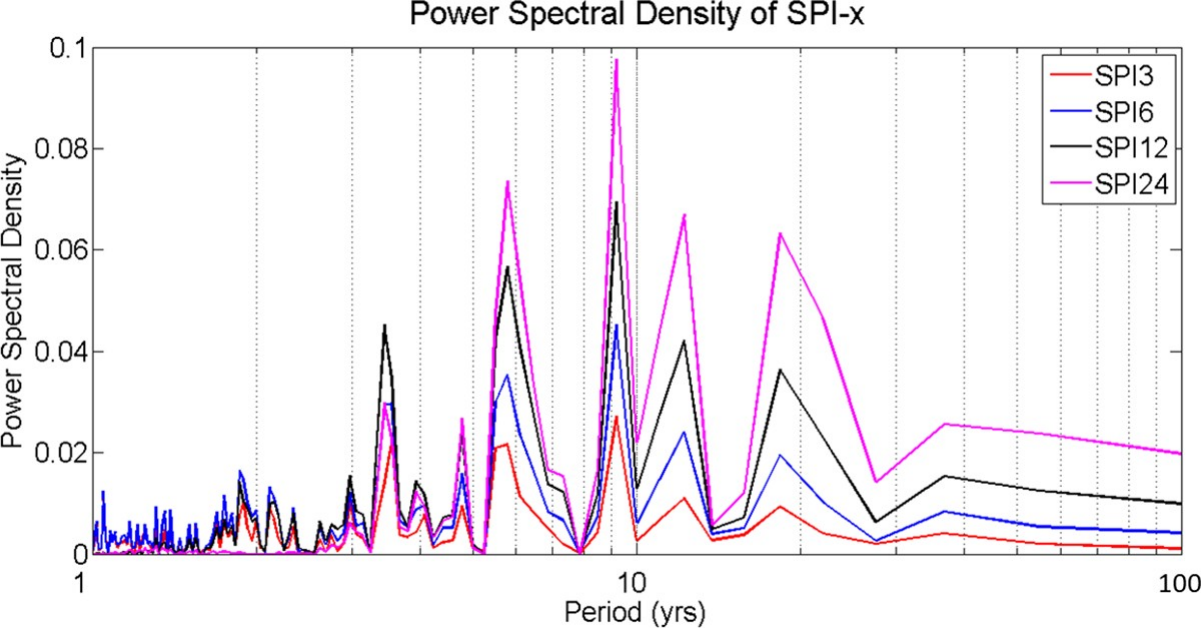
Power spectral density of the SPI-3, -6, -12, and -24 values.

Application of the Fourier transformation requires a detrended input data. SPI is an index value with mean zero and standard deviation of one, which permits its use in this analysis. Droughts that persist between 3 and 6 months (SPI-3 and SPI-6) have 2 to 3 years of the cycle, while droughts that persist between 1 and 2 years (SPI-12 and-24) have 3 different frequency ranges (5 to 6, 9 to 10, and 10 to 20) (Fig 12).

### Implications for freshwater availability

The impact of hydroclimate variability on the freshwater availability in Southeast Florida is described based on the implications of increasing rainfall intensity and incidence of drought. Freshwater in the area is stored in lakes, the Everglades wetland, and the aquifer systems. The man-made modifications in the area have amplified the high natural hydrologic connectivity among different reservoirs. In general, surface water reservoirs have quick response to stress conditions, however, in Southeast Florida, the groundwater and surface water system is highly connected [1,2]. As a result, the feedback of the entire hydrologic system have quick response to any sustained stress or pulses.

The Biscayne Aquifer (BA) is highly dependent on recharge from the Everglades. For example, the rainfall decline of 30 inches (762 mm) due to drought in 1980 to 1982 caused a significant decline in the water level at Lake Okeechobee, the Everglades, flow along canals, and groundwater head in the BA [1,2]. Rainfall deficit increases irrigation water demand, increases the dependence on groundwater pumping, leading to groundwater head decline in the BA.

**Fig 13:**
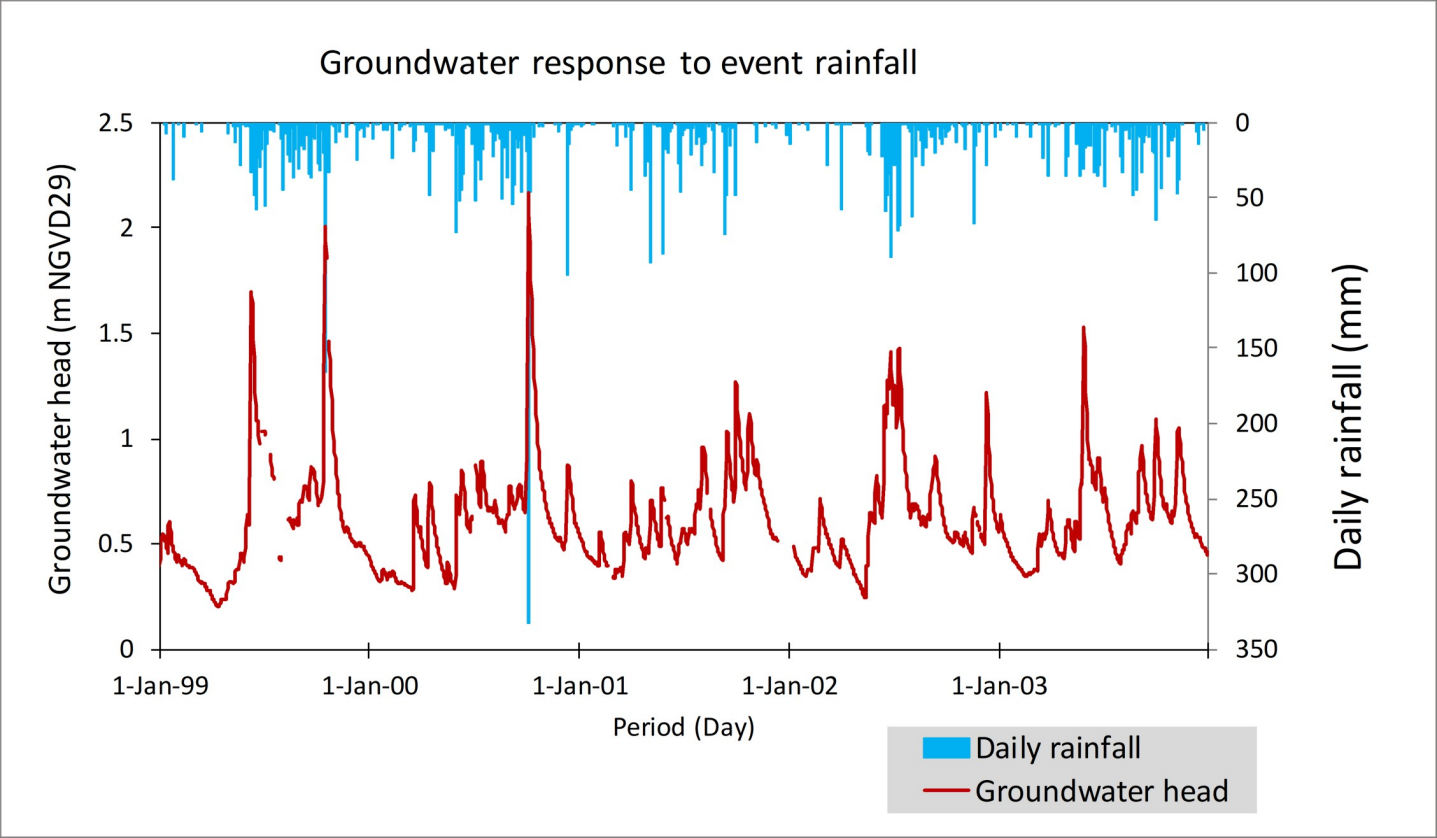
The quick response of the Biscayne Aquifer groundwater table to event rainfall. The rainfall and groundwater measurement are collected from a station close to S331W (Fig 14).

The BA is highly permeable karstic system with the mean hydraulic conductivity of 9,000 m/d that has a rapid response to rainfall recharge (Fig 13). Conversely, the groundwater table is highly elastic to hydrological stress conditions [1], although, a decline in recharge reduces in the underlining aquifer’s hydraulic pressure, increasing the hydraulic gradient along the coastal boundaries and the potential for saltwater intrusion into the freshwater system.

**Fig 14:**
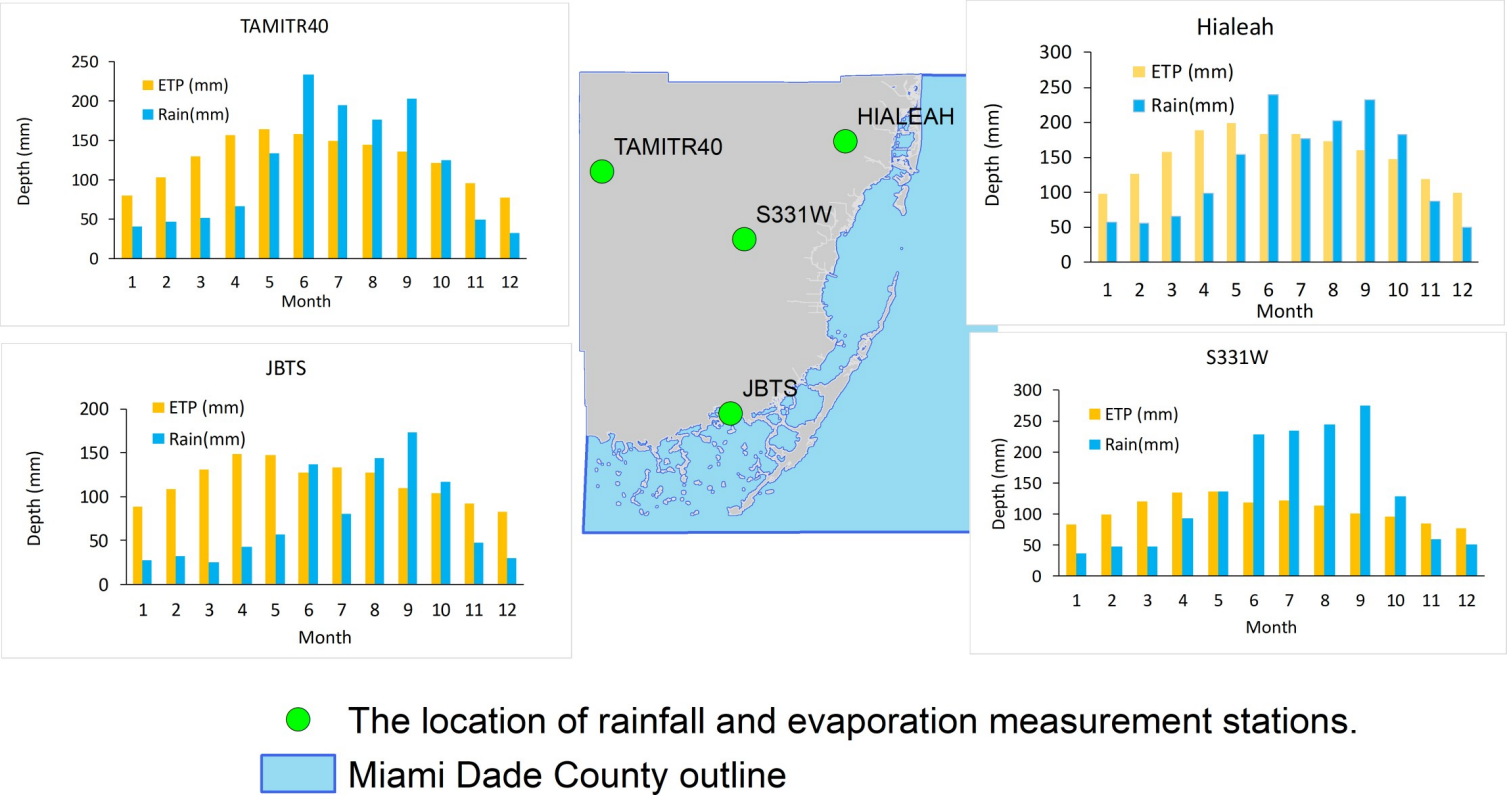
Rainfall and ET record comparison between the coastal and Everglades sides of the study area. JBTS and Hialeah stations represent the coastal region, and TAMITR40 is located in the Everglades. S331W represents the central region of the study area.

Evaporative loss is another threat to freshwater sustainability in the Everglades. The Everglades have year-round evaporation that is higher than the rainfall it receives (Fig 14). Although the urban side has a positive balance, the net water balance is profoundly affected by groundwater pumping.

The other perspective of hydroclimate variability in the area is the impact of decreasing wet season duration. The decreasing wet season duration can cause a decrease in groundwater recharge. The elongation of the dry season increases water user’s dependence on water supply from storage and groundwater pumping. To better address such drought-driven freshwater availability deficits, appropriate water conservation practice is necessary.

Evaluation and selection of best water conservation practices to enhance the self-resilience of the BA will decrease the dependence of the BA on recharge from the Everglades. To reduce hydrological stress on the Everglades, water management methods to increase freshwater head in the Everglades, promote groundwater recharge into the BA, and limit the groundwater demand in the area can decrease the susceptibility of freshwater supply to short and long-term hydrological stress conditions.

Promoting high freshwater head and expanding water storage mechanisms in the Everglades are promising hydrological water management options. This requires constructing and expansion of water storage that can enhance freshwater flow to the Everglades. The hydrological management measures benefit both the Everglades Ecosystem and water supply infrastructures. Conservation practices that discourage groundwater pumping can be of practical importance to maintain adequate groundwater head. In the face of high uncertainty to drought predictions, water resources planning in Southeast Florida shall consider multiple scenarios commensurate to different levels of stress conditions. However, an in-depth evaluation of the fate of the BA in the face of prologue drought and saltwater intrusion is a substantial input to review the existing water supply management system.

## Conclusion and recommendation

From the analyses of the trend of monthly rainfall records from 1906 to 2016, results indicate increase in the total wet season, the mean annual, and total annual rainfall. The wet season duration is decreasing due to a decrease in October rainfall. Rainfall in August is increasing, promoting a gradual shift of the local rainfall pattern from bimodal to a unimodal regime. The emergence of unimodal rainfall regime, decreasing wet season duration, and an increased total annual rainfall suggest the increasing importance of freshwater storage and water harvesting in the area.

Given the persistent positive values of the SPI-12 and SPI-24 since 1994/5, Southeast Florida is in the wet phase of the long-term hydroclimate variabilities. The area is receiving rainfall much above the long-term average. Drought frequency analysis indicates that short-ranged drought events have 3- to 5-years cycle, and sustained drought has a cycle of 10- to 20-years. Thus, Southeast Florida is prone to a minimum of one small-scale drought every three years and a minimum of one large-scale drought every ten years.

With the increasing dry season duration, it is possible that small-scale droughts can propagate over a longer period. Prolonged small-scale drought tends to accumulate its effect gradually and unnoticed, and eventually compromising the sustainable water resource system. This can affect freshwater availability in the Everglades, disrupting the local ecosystem and ecosystem services.
